# 烟台和威海近海医药与个人护理用品分布、多介质分配行为及生态风险

**DOI:** 10.3724/SP.J.1123.2024.10023

**Published:** 2025-08-08

**Authors:** Han CHEN, Shuang LU, Zixuan YU, Yixuan MA, De WANG, Zhihua SONG, Min LYU, Lingxin CHEN, Jing DING

**Affiliations:** 1.烟台大学环境与材料工程学院，山东 烟台 264005; 1. School of Environmental and Materials Engineering，Yantai University，Yantai 264005，China; 2.中国科学院烟台海岸带研究所，山东 烟台 264003; 2. Yantai Institute of Coastal Zone Research，Chinese Academy of Sciences，Yantai 264003，China; 3.烟台市海洋生态环境核安全保障重点实验室，山东 烟台 264003; 3. Yantai Key Laboratory of Nuclear Safety Assurance for Marine Ecological Environment，Yantai 264003，China

**Keywords:** 医药与个人护理用品, 时空分布, 沉积物-水分配系数, 生态风险评价, pharmaceuticals and personal care products（PPCPs）, spatial-temporal distribution, sediment-water partitioning coefficient, ecological risk assessment

## Abstract

为全面评估医药与个人护理用品（PPCPs）的分布、多介质分配行为及影响其环境行为的关键环境因素，本研究采用野外采样和室内高精度分析相结合的方法，全面系统探究了15种非甾体类消炎药和防腐剂在烟台和威海近海水及沉积物中的时空分布、分配行为及生态风险。结果显示，海水和沉积物中15种物质质量浓度分别为2.31~662.31 ng/L和57.11~31.09 ng/g，安替比林（PHZ）、非诺洛芬（FPF）、对羟基苯甲酸甲酯（MPB）和布洛芬（IBU）、酮洛芬（KPF）分别为海水和沉积物中的主要污染物。PPCPs浓度在夏季较高，这与其使用方式及夏季旅游城市的高强度人类活动密切相关。夏季MPB、IBU、PHZ和FPF的平均质量浓度分别可达30.16、15.15、3.27和3.81 ng/L。空间分布上，春季和冬季渤海海域采样点PPCPs浓度高于黄海海域，夏季黄海海域采样点PPCPs浓度要高于渤海海域，而表底层海水中目标物质总浓度未呈现出显著性差异；沉积物中PPCPs浓度为黄海高于渤海。对羟基苯甲酸乙酯和对羟基苯甲酸丙酯的沉积物-水分配系数分别为（3.99±0.95）和（3.80±0.57） cm^3^/g，高于EPISuite模型的预测值，表明实际环境中污染物在沉积物-水间分配行为的复杂性。生态风险方面，总风险熵值结果表明，PPCPs混合物对研究区海水中藻类、甲壳类和鱼类呈低风险。尽管未呈现出显著差异，PPCPs混合物对甲壳类动物的风险熵值高于藻类和鱼类。为明确研究区海域中是否存在具有高风险的优先控制PPCPs，计算了单一PPCP的优先指数（PI），结果表明所有PPCPs的PI值均为零，因此被归类为安全污染物。但在未来的研究中，仍需要不断关注海洋环境中多种PPCPs的浓度水平和风险。

## Abstract (en)

To comprehensively assess the distribution， multi-media-partitioning behavior， and key environmental factors that influence the environmental fates of pharmaceuticals and personal care products（PPCPs）， this study employed a combination of field sampling and high-precision analysis to systematically investigate the spatiotemporal distributions， partitioning behavior， and ecological risks of 15 nonsteroidal anti-inflammatory drugs（NSAIDs） and preservatives in the seawater and offshore sediments of Yantai and Weihai. The results showed that the total mass concentrations of the 15 compounds ranged between 2.31 and 662.31 ng/L in seawater， and between 57.11 and 131.09 ng/g in sediments. Among them， antipyrine（PHZ）， fenoprofen（FPF）， and methylparaben（MPB） were the major pollutants in seawater， whereas ibuprofen（IBU） and ketoprofen（KPF） were dominant in sediments. Higher concentrations of PPCPs were observed in the summer， which closely mirrors their usage patterns and human activities during the summer and peak tourist seasons in Yantai and Weihai City. MPB， IBU， PHZ， and FPF exhibited average mass concentrations of 30.16， 15.15， 3.27， and 3.81 ng/L， respectively， in the summer. Regarding the spatial distributions of these compounds， the seawater collected from the Bohai Sea exhibited a higher total PPCP concentration than that in the Yellow Sea in spring and winter， whereas the reverse was observed in summer. Additionally， the total PPCP concentrations in surface and bottom seawater showed no significant seasonal differences. Sediment in the Yellow Sea was found to contain a higher total PPCP concentration than that in the Bohai Sea sediment. Ethylparaben and propylparaben exhibited sediment-water partitioning coefficients of（3.99±0.95） and（3.80±0.57） cm^3^/g， respectively， which are higher than their corresponding theoretically predicted values from EPISuite model， indicating the complexity of pollutant partitioning behavior between sediment and water in real environments. In terms of ecological risks， total risk quotient（RQ_T_） values revealed that PPCP mixtures are likely to pose low risks to algae， crustaceans， and fish in the study area. In addition， while crustaceans exhibited a higher RQ_T_ value for PPCP mixtures than algae or fish， the differences were not significant. To identify whetherigh-risk pollutants existed in the study area， the prioritization index（PI） of each individual PPCP was calculated. The target PPCPs were found to have PI values of zero and were consequently classified as safe pollutants. Nonetheless， continuous attention needs to be paid to the levels and risks associated with multiple PPCPs in the marine environment， in view of the relative scarcity of studies.

医药与个人护理用品（PPCPs）作为与人类生活密切相关的新污染物，受到国内外广泛关注。PPCPs主要包括用于人类和动物疾病治疗或预防的药物以及用于个人护理品中的消毒剂、防腐剂、防晒剂等。其中，非甾体类消炎药（non-steroid anti-inflammatory drug，NSAIDs）是一类常用于治疗关节炎、免疫系统疾病和发热疼痛等的药物，因其显著的消炎、镇痛作用而被广泛应用于临床；研究显示，全球每天有超过3 000万人服用NSAIDs^［1］^。对羟基苯甲酸酯类可抑制微生物活动^［2］^，是一类广泛用于药物和个人护理产品中的防腐剂^［3］^。

消炎药和防腐剂因使用量大，被持续排放到环境中，造成其在包括美国旧金山湾^［4］^、沙特阿拉伯红海^［5］^、中国厦门湾^［6］^和渤海^［7］^在内的国内外海水中广泛检出。研究发现，不同种类消炎药和防腐剂的检出频率具有较大差异，例如，渤海水体中安替比林（PHZ）、异丙安替比林（PPZ）、布洛芬（IBU）和酮洛芬（KPF）的检出频率均为100%，而萘普生（NPX）、吲哚美辛（IND）、非诺洛芬（FPF）的检出频率为0~43.5%^［7］^。与传统持久性有机污染物相比，消炎药和防腐剂具有相对较短的半衰期，在海水中易被光降解和生物降解^［8］^。考虑到污染物降解受环境条件影响，季节和空间变化引起的环境条件改变、不同物质的消费模式变化会导致PPCPs分布特征发生改变。此外，随着医疗卫生条件的改善，药品消费量增大，也改变了PPCPs的消费模式和数量^［9］^，进而导致水环境中PPCPs的赋存和时空分布存在区域特异性。值得注意的是，虽然PPCPs在海水中的质量浓度通常为几ng/L到几十ng/L，但低浓度持续暴露会对水生生物产生不同程度的生态毒性效应，甚至危害其生命。因此，探究不同海域中PPCPs的赋存特征，评估其对水生生物的生态风险，对于揭示PPCPs污染过程和环境行为具有重要意义。

海洋沉积物是各类污染物的重要储存库，对海洋环境中污染物分布、迁移和归趋具有重要影响。海水中的部分PPCPs会经水动力扩散或随悬浮颗粒沉降进入沉积物，沉积物受底层水冲刷后，沉积物-水界面的PPCPs再悬浮至上覆水中，影响水体PPCPs分布和垂向迁移，进而导致PPCPs的赋存特征呈现时空变异性。然而，目前国内外对于海洋沉积物中PPCPs赋存特征的研究较为匮乏，对于PPCPs在沉积物-水界面的分配行为更是鲜少报道。因此，为深入了解PPCPs在环境中的归趋，亟待探究海洋环境中PPCPs的多介质分配行为。

近海蕴藏丰富的海洋资源，也是各种污染物的最终汇集地，生态环境健康面临巨大威胁。烟台和威海海域（以下简称烟威海域）位于环渤海经济区内，区域内人口密度高，旅游资源丰富。高强度的人类活动可能导致烟威海域非甾体类消炎药和防腐剂的污染状况较其他区域更为严重。以往对该地区的研究大多集中于重金属^［10，11］^和海洋生物^［12，13］^，对该地区海水和沉积物中非甾体类消炎药和防腐剂时空分布特征、多介质分配行为及生态风险的系统研究尚未深入开展，且该海域是否存在需重点关注的高风险物质尚不明确。因此，本研究以黄渤海近岸烟威海域为研究区，于不同季节采集表层海水、底层海水和沉积物样品，对海水和沉积物中15种非甾体类消炎药和防腐剂的浓度水平和分布特征进行深入探究，阐明目标污染物在沉积物-水之间的分配行为，并系统评估PPCPs混合物长期存在对不同营养水平水生生物的生态风险，识别具有高风险的优先控制污染物。研究结果可丰富近海PPCPs环境行为理论体系，为新污染物管控的国家需求提供科学依据。

## 1 实验部分

### 1.1 研究区概况和样品收集

研究区位于山东半岛北部黄海与渤海交界处烟威海域，属于温带季风气候。根据沿岸主要城市分布情况，本研究共设置4个采样断面，共14个采样点。其中A1～A4位于龙口与莱州交界海域，B1～B3位于蓬莱海域，C1～C4位于烟台海域，D1～D3位于威海海域（图1）。

**图1:**
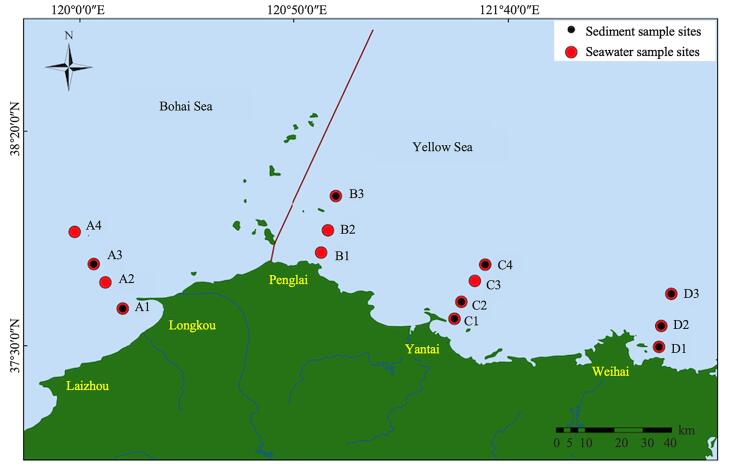
研究区域及采样点位 The figure was generated using ArcGIS 10.8.

本研究分别于2022年5月（春季）、2022年7月（夏季）和2023年2月（冬季）使用CTD采水器在14个采样点位采集表层海水（海平面下约0.5 m）和底层海水（海底上方1 m），共采集海水样品83个（受采样现场实际环境条件影响，冬季未在D3点位采集底层海水）。此外，在春季使用箱式采泥器于9个点位采集表层沉积物（0~2 cm）样品（图1）。水样采集后放置于清洗好的棕色玻璃瓶中，经过滤（GF/F，0.7 µm，Whatman，USA）和盐酸（0.1 mol/L）酸化（pH 2~3）后于4 ℃下黑暗环境中运输到实验室并尽快进行后续处理。沉积物样品经采集后用铝箔包装，密封后于-18℃冷冻条件下储存。采样现场同步监测水样盐度、电导率和pH值。

### 1.2 仪器与试剂

超高效液相色谱-三重四极杆质谱仪（Applied Biosystems， Framingham，美国）。CTD采水器和Ekman箱式采泥器（KC-Denmark，丹麦）；HLB固相萃取柱（500 mg，6 mL，Waters，美国）。

本研究选取使用量较大的9种非甾体类消炎药和6种防腐剂作为目标物质（表1）。15种PPCPs和同位素标准品（对羟基苯甲酸甲酯-^13^C_6_，MPB-^13^C_6_）纯度均高于98%，购自Dr. Ehrenstorfer 公司（Augsburg，Germany）、AccuStandard（New Haven，United States）、Fluka（St. Louis，United States）或Sigma-Aldrich（Flanders，USA）。实验所用甲酸和丙酮均为色谱纯，购自德国默克公司。实验所用水均为超纯水（>18.2 MΩ/cm），取自Millipore制水系统（USA）。

**表1:** 本研究15种PPCPs清单

Type	Compound	Abbreviation	CAS No.	Molar mass/（g/mol）	Solubility^［8］/^（mg/L）
Non-steroid anti-inflammatory drug（NSAIDs）	ibuprofen	IBU	15687-27-1	206.3	2.10×10
	paracetamol	PTL	103-90-2	151.2	1.40×10^4^
	propyphenazone	PPZ	479-92-5	230.3	2.40×10^3^
	ethenzamide	EZM	938-73-8	165.2	1.41×10^4^
	phenazone	PHZ	61-68-7	241.3	5.19×10^4^
	naproxene	NPX	22204-53-1	230.3	1.59×10
	indomethacine	IND	53-86-1	357.8	9.37×10^-1^
	fenoprofen calcium dihydrate	FPF	53746-45-5	522.6	3.01×10
	ketoprofen	KPF	22071-15-4	254.3	5.10×10
Preservative	methyl paraben	MPB	99-76-3	152.2	2.50×10^3^
	ethyl paraben	EPB	120-47-8	166.2	8.85×10^2^
	propyl paraben	PPB	94-13-3	180.2	5.00×10^2^
	butyl paraben	BUT	94-26-8	194.2	2.07×10^2^
	heptyl paraben	HPB	1085-12-7	236.3	2.00×10
	benzyl paraben	BPBen	94-18-8	228.3	1.08×10^2^

### 1.3 样品预处理和目标PPCPs分析测定

样品经预处理流程后，采用超高效液相色谱-三重四极杆质谱仪测定样品中目标PPCPs含量^［8］^。具体步骤如下：海水样品（1 L）经过滤和酸化后，加入500 mg Na_4_EDTA和30 ng同位素标准品。随后用已活化（20 mL甲醇、6 mL超纯水和6 mL pH为2~3的酸化水）的HLB固相萃取柱提取PPCPs，并先后用12 mL甲醇和6 mL甲醇-丙酮（50∶50，v/v）混合液进行洗脱。洗脱液经氮吹至近干，并用甲醇定容至1.0 mL后待测。沉积物经冷冻干燥、研磨过筛后，取2 g置于50 mL离心管中，加入30 ng同位素标准品。之后加入10 mL EDTA-McΙlvaine缓冲液（pH 4.0，采用柠檬酸、磷酸氢二钠和乙二胺四乙酸二钠配制），涡旋1 min，再加10 mL乙腈，涡旋1 min，超声30 min。加入4 g硫酸镁和1 g氯化钠，立即强力振摇3 min，离心，取7 mL上清液转移至棕瓶，氮吹至近干，并用甲醇定容至1.0 mL后待测。

色谱条件 BEH C18色谱柱（100 mm×2.1 mm， 1.7 μm， Waters），保护柱为EVO C_18_（5 mm×2.1 mm，1.7 μm，Phenomenex）。柱温35 ℃，流动相流速0.3 mL/min，进样体积10 μL。以0.01%甲酸水（A）和甲醇（B）为流动相，采用梯度洗脱：0~1.0 min，5%B；1.0~2.0 min，5%B~35%B；2.0~6.0 min，35%B~50%B；6.0~13.0 min，50%B~90%B；13.0~14.0 min，90%B~100%B；14.0~16.0 min，100%B；16.0~16.1 min，100%~5%B；16.1~18.0 min，5%B。

质谱条件 电喷雾电离源（ESI^+^/ESI^-^），多反应监测（MRM）模式，具体参数见表2。

**表2:** 目标PPCPs和内标物质的质谱参数

Compound	Parent ion（*m/z*）	Daughter ions^a ^（*m/z*）	DPs/V	EPs/V	CEs/eV	CXPs/V	Mode
IBU	205.0	159.0/161.0	-50	-10	-9/-12	-2	ESI^-^
PTL	152.0	110.0	61	4.5	21	4	ESI^+^
PPZ	231.0	56/189	86	11	49/38	4	ESI^+^
EZM	166.0	121.0/149.0	31	9.0	25/15	4	ESI^+^
PHZ	189.0	77.3/146.6	61	4.5	40/30	4	ESI^+^
NPX	229.1	169.0/185.0	-40/-30	-7.5	-41/-15	-2	ESI^-^
IND	358.0	139.0/174.0	61	4.5	27/19	4	ESI^+^
FPF	241.0	93.0/196.9	-13/-15	-3.0	-47/-12	-3/-7	ESI^-^
KPF	253.1	197.0/209.0	-26	-5/-8	-10/-10	-9	ESI^-^
MPB	150.9	91.3	-49	-10	-28	-10	ESI^-^
EPB	165.1	137.0/92.1	-92/-94	-10	-18/-31	-7	ESI^-^
PPB	178.9	136.0/91.8	-102/-98	-7.5	-21/-30	0	ESI^-^
BUT	192.9	92.1/136.0	-94	-6.0	-32/-23	-6/-4	ESI^-^
HPB	235.0	137.0/92.1	-109	-10	-25/-41	-4	ESI^-^
BPBen	227.0	136.0/92.0	-100/-97	-2.0/-3.0	-21/-31	-4/-6	ESI^-^
MBP-^13^C_6_	157.0	98.1	-49	-10	-28	-10	ESI^-^

a： quantitative ion/confirmation ion； DP： declustering potential； EP： entrance potential； CE： collision energy； CXP： collision cell exit potential.

### 1.4 质量保证和质量控制

本研究遵循严格的质量控制和保证程序。为保证实验数据的可靠性，每个批次设置一个过程空白、一个空白加标、一个基质加标和一个平行样品，且每个样品在提取前均加入同位素标准品指示回收率。为核查仪器稳定性，每20个样品插入一个已知浓度标准样品，并将已知浓度标准样品的含量偏差控制在20%的范围内。目标物质的同位素标准品回收率在50%~120%内，且相对标准偏差小于15%。此外，分别以3倍和10倍信噪比确定目标物质的方法检出限（MDL）和方法定量限（MQL）^［7］^，海水和沉积物样品中目标物质的MDL范围分别为0.03~2.00 ng/L和0.02~0.85 ng/g，目标物质的MQL范围分别为0.20~2.23 ng/L和0.12~1.63 ng/g。

### 1.5 生态风险评估及优先控制PPCPs识别

自然水体中的PPCPs通常以混合物的形式存在，它们在水生环境中的风险不会单独发生，忽视混合物风险可能会低估对水生生物的实际影响^［14］^。为评估多种PPCPs残留造成的生态风险，本研究基于浓度叠加模型^［15］^评估了PPCPs混合物对于藻类、甲壳类和鱼类的总风险熵值RQ_T_（total risk quotients）：


$$RQ_{T}=\sum_{i=1}^{n}RQ_{i}=\sum_{i=1}^{n}\frac{MEC_{i}}{PNEC_{i}}$$ (1)

其中，MEC *_i_* （measured environmental concentration of compound *i*）为化合物*i*在海水中的实测浓度，PNEC *_i_* （predicted no-effect concentration）为化合物*i*的预测无效应浓度，RQ *_i_* 为化合物*i*的生态风险熵值。风险熵值越高，表明其生态风险等级越高。根据风险熵值的大小，将生态风险等级分为高风险（RQ_T_≥1）、中风险（0.1≤RQ_T_<1）和低风险（RQ_T_<0.1）^［8，15-17］^。

目标PPCPs预测无效应浓度的计算是基于以生长或繁殖异常为终点（endpoints）的慢性毒性数据——无观察效应浓度（no observed effect concentration， NOEC）或最低观察效应浓度（lowest observed effect concentration， LOEC）和相应评估因子（assessment factor， AF）^［8，14］^，计算公式如下：


$$PNEC=\frac{NOEC\;or\;LOEC\;}{AF}\;$$ （2）

其中，目标PPCPs对藻类、甲壳类和鱼类的 NOEC /LOEC值收集自美国环境保护局ECOSAR v2.2软件。当3个营养级生物均有可用慢性毒性数据时，AF值取10^［8］^；此外，当同一营养级生物有多个可用毒性数据时，选择可用毒性数据的最低值计算PNEC，以实现对最敏感物种的最大程度保护。收集得到目标PPCPs对不同水生生物的慢性毒性数据和计算得到的预测无效应浓度见表3。

**表3:** 目标PPCPs对不同水生生物的慢性毒性数据和预测无效应浓度

Compound	Taxonomic group	Endpoint	Toxic data/（µg/L）	Toxicity	AF	PNEC/（ng/L）
BUT	algae	NOEC	2.30×10^3^	chronic	10	2.30×10^5^
BUT	crustaceans	NOEC	7.06×10^2^	chronic	10	7.06×10^4^
BUT	fish	LOEC	8.76×10^2^	chronic	10	8.76×10^4^
BPBen	algae	LOEC	1.95×10^3^	chronic	10	1.95×10^5^
BPBen	crustaceans	NOEC	5.55×10^2^	chronic	10	5.55×10^4^
BPBen	fish	NOEC	6.50×10^2^	chronic	10	6.50×10^4^
EZM	algae	LOEC	8.23×10^4^	chronic	10	8.23×10^6^
EZM	crustaceans	LOEC	6.17×10^4^	chronic	10	6.17×10^6^
EZM	fish	NOEC	1.46×10^5^	chronic	10	1.46×10^7^
EPB	algae	NOEC	7.68×10^3^	chronic	10	7.68×10^5^
EPB	crustaceans	LOEC	3.27×10^3^	chronic	10	3.27×10^5^
EPB	fish	LOEC	5.13×10^3^	chronic	10	5.13×10^5^
FPF	algae	NOEC	1.57×10^4^	chronic	10	1.57×10^6^
FPF	crustaceans	NOEC	4.19×10^3^	chronic	10	4.19×10^5^
FPF	fish	LOEC	4.68×10^3^	chronic	10	4.68×10^5^
HPB	algae	LOEC	3.61×10^2^	chronic	10	3.61×10^4^
HPB	crustaceans	LOEC	6.80×10^1^	chronic	10	6.80×10^3^
HPB	fish	LOEC	5.90×10^1^	chronic	10	5.90×10^3^
IBU	algae	LOEC	1.56×10^4^	chronic	10	1.56×10^6^
IBU	crustaceans	NOEC	4.31×10^3^	chronic	10	4.31×10^5^
IBU	fish	NOEC	4.94×10^3^	chronic	10	4.94×10^5^
IND	algae	NOEC	1.48×10^4^	chronic	10	1.48×10^6^
IND	crustaceans	NOEC	3.54×10^3^	chronic	10	3.54×10^5^
IND	fish	NOEC	3.66×10^3^	chronic	10	3.66×10^5^
KPF	algae	NOEC	5.77×10^4^	chronic	10	5.77×10^6^
KPF	crustaceans	LOEC	2.07×10^4^	chronic	10	2.07×10^6^
KPF	fish	LOEC	2.88×10^4^	chronic	10	2.88×10^6^
MPB	algae	LOEC	1.39×10^4^	chronic	10	1.39×10^6^
MPB	crustaceans	LOEC	6.96×10^3^	chronic	10	6.96×10^5^
MPB	fish	LOEC	1.23×10^4^	chronic	10	1.23×10^6^
NPX	algae	LOEC	4.53×10^4^	chronic	10	4.53×10^6^
NPX	crustaceans	NOEC	8.20×10^4^	chronic	10	8.20×10^6^
NPX	fish	NOEC	2.13×10^4^	chronic	10	2.13×10^6^
PPZ	algae	NOEC	1.96×10^4^	chronic	10	1.96×10^6^
PPZ	crustaceans	NOEC	9.64×10^3^	chronic	10	9.64×10^5^
PPZ	fish	LOEC	1.68×10^4^	chronic	10	1.68×10^6^
PHZ	algae	LOEC	1.21×10^5^	chronic	10	1.21×10^7^
PHZ	crustaceans	LOEC	9.62×10^4^	chronic	10	9.62×10^6^
PHZ	fish	NOEC	2.38×10^5^	chronic	10	2.38×10^7^
PPB	algae	NOEC	4.21×10^3^	chronic	10	4.21×10^5^
PPB	crustaceans	LOEC	1.52×10^3^	chronic	10	1.52×10^5^
PPB	fish	LOEC	2.13×10^3^	chronic	10	2.13×10^5^
PTL	algae	LOEC	1.52×10^5^	chronic	10	1.52×10^7^
PTL	crustaceans	NOEC	1.35×10^5^	chronic	10	1.35×10^7^
PTL	fish	NOEC	3.61×10^5^	chronic	10	3.61×10^7^

AF： assessment factor；PNEC： predicted no-effect concentration；NOEC： no observed effect concentration； LOEC： lowest observed effect concentration.

此外，为有效识别出具有高生态风险的优先控制PPCPs，本研究基于优化的风险熵值法计算了每种物质的优先级指数PI^［8，9，15，17］^：


$$PI_{i}=RQ_{Mi}\times \left(\frac{N_{1}}{N}\times 100\%\right)$$ （3）

其中，RQ_M_ *_i_* （mean risk quotient value of compound *i*）为化合物*i*对其最敏感物种的平均风险熵值，即根据3个营养级（藻类、甲壳类和鱼类）中最低的PNEC计算。*N*_1_为浓度高于PNEC *_i_* 的样本数，*N*为总样本数（*N*=83）。根据PI的数值，可将目标化合物分为高风险（PI≥1）、中风险（0.1≤PI<1）低风险（0.01≤PI<0.1）、可忽略风险（0<PI<0.01）和安全（PI=0）^［8，15，17］^。

### 1.6 统计分析

本研究主要采用SPSS 27软件进行数据检验，在显著性水平0.05下检验统计学显著性和Spearman相关性。采用Origin 2022绘制箱线图，采用ArcGIS软件（v10.8）分析非甾体类消炎药和防腐剂的空间分布特征。当物质浓度小于方法检出限时，使用Excel中的任意赋值功能（RANDBETWEEN）将物质浓度赋值为0~LOD之间的任意数值以进行数据统计分析^［18］^。

## 2 结果与讨论

### 2.1 海水和沉积物中目标PPCPs的检出频率和浓度水平

目标PPCPs的检出频率和浓度水平如图2所示。除扑热息痛（PTL）和PPZ外，其余13种PPCPs在海水和沉积物中均被检出（图2a）。其中，PHZ、FPF、对羟基苯甲酸甲酯（MPB）、对羟基苯甲酸乙酯（EPB）和对羟基苯甲酸丙酯（PPB）在海水中的检出频率为70%~100%；IBU、KPF、MPB、EPB、PPB在沉积物中的检出频率均高于50%。整体上，PPCPs的检出频率与之前临近区域的检出频率相当或略低^［8，15］^，表明目标PPCP在研究区普遍存在。

**图2:**
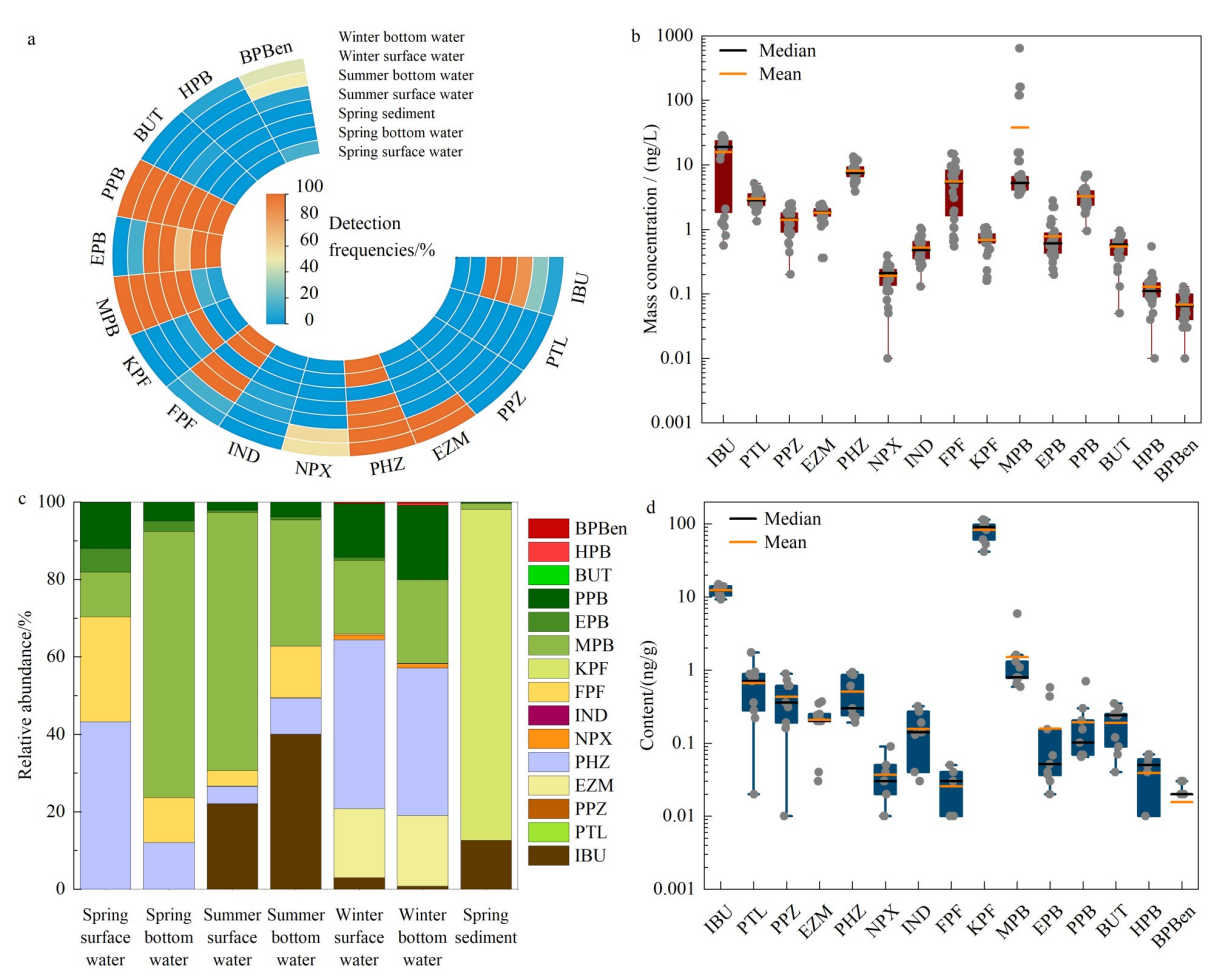
目标PPCPs（a）在海水和沉积物中的检出频率、（b）在海水中的质量浓度、（c）在海水和沉积物中的相对丰度以及（d）在沉积物中的含量

海水中PPCPs总质量浓度范围为2.31~662.31 ng/L，PHZ、FPF和MPB是海水中主要的PPCPs（图2b、2c）。整体上，烟威海域采样点海水中PPCPs的浓度平均值低于或与世界其他区域海水中目标物的浓度水平相当^［5，19-21］^。前期研究表明PHZ是渤海海域主要的PPCPs污染物，PHZ、FPF和MPB对总PPCPs浓度的贡献率为55%~92%^［7］^。本研究中，MPB分别占春季底层水和夏季表层水中PPCPs总浓度的69%和67%（图2c），这可能与其在我国市场上占据主导地位有关^［3］^。然而，以往关于烟威海域的研究中未涉及MPB^［8，22，23］^，因此可能低估了MPB所导致的防腐剂污染。PHZ在世界范围内均已被检测到^［8，24-26］^，本研究中PHZ的质量浓度（平均值：3.27 ng/L）与烟台湾（平均值：3.40 ng/L）^［8］^、德国-荷兰跨界Vecht河（平均值：2.09×10^-2^ ng/L）相当，低于荷兰莱茵河（平均值：135 ng/L）^［26］^、东地中海沿岸（西班牙）米哈雷斯河（最大值：1 984 ng/L）^［27］^；FPF的质量浓度（平均值：3.81 ng/L）高于烟台湾（最大值：0.09 ng/L）^［8］^；MPB的质量浓度（平均值：30.16 ng/L）低于巴西（最大值：980 ng/L）^［28］^，高于渤海（平均值：3.3 ng/L）^［7］^。

沉积物中PPCPs的总含量范围为57.29~126.34 ng/g（图2d）。不同于海水，沉积物中非甾体类消炎药的浓度整体高于防腐剂（图2d），值得注意的是，海水中的主导物质PHZ在沉积物中的浓度低于其检出限（图2d），可能与其在水中较高的溶解度有关；相反地，IBU和KPF在海水中的浓度低于其检出限，但在沉积物中具有较高浓度，这可能与这两种物质的高log *K*_ow_值（分别为3.72和2.81）有关。特别地，KPF是沉积物中浓度最高的污染物。本研究中IBU含量（平均值：12.31 ng/g）甚至高于北运河沉积物（平均值：ND~0.46 ng/g）^［29］^，但低于地中海沿岸湿地沉积物中的IBU含量（最大值：100 ng/g）^［30］^；长江口沉积物中检测到了IND（含量范围：12~164 ng/g）^［31］^，但本研究区域沉积物中并未检测到IND；本研究区域MPB与地中海沿岸湿地的含量相当（平均值低于10 ng/g）^［30］^。

### 2.2 海水中目标PPCPs浓度的季节变化

PPCPs浓度呈现季节性差异。非甾体类消炎药、防腐剂和PPCPs总浓度整体表现为夏季高于春季和冬季（图3）。夏季防腐剂的高浓度主要与MPB和PPB在夏季的较高浓度有关，其平均质量浓度分别为30.16 ng/L和1.45 ng/L。与其他两个季节相比，夏季消费者为维持自身卫生状况和抵御紫外线伤害，日常生活中个人洗护产品用量增大，进而导致防腐剂在夏季的排放量增加。此外，夏季沿海城市旅游人口的增长同样是导致防腐剂排放量增加的潜在因素。已有研究报道夏季丰水期时，较强的旅游活动导致防腐剂和防晒剂在九龙江入海口具有较高的浓度^［32］^。本研究区域夏季MPB的质量浓度要远高于夏季新西兰利特尔顿港（<2.7 ng/L）^［33］^和葡萄牙阿威罗地区海水（平均值：9.9 ng/L）^［34］^的浓度水平，而PPB浓度与上述研究区域浓度水平相当^［33，34］^。

**图3:**
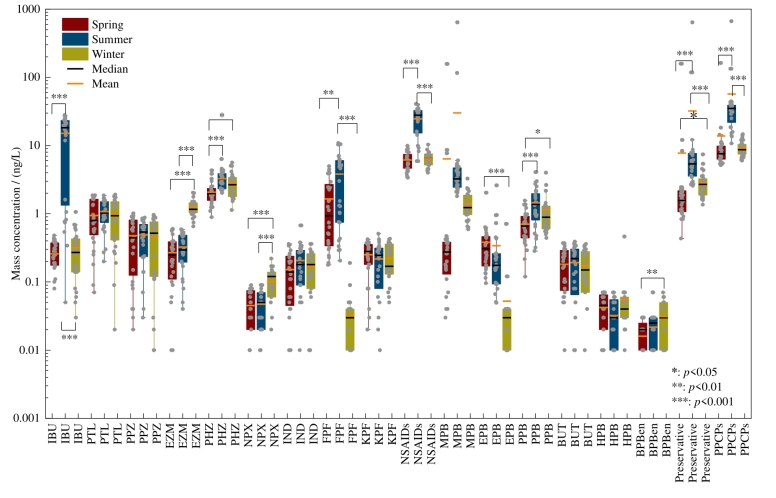
海水中目标PPCPs质量浓度的季节性变化

主要非甾体类消炎药IBU、PHZ、FPF在夏季具有较高的浓度，平均质量浓度分别为15.15、3.27和3.81 ng/L（图3）。其中，IBU只在夏季样品中被检测到，FPF在春季和夏季样品中被检测到，PHZ在3季样品中都被检测到。IBU是一种源自丙酸的非甾体类消炎药，是最常用的非处方药之一，在我国七大流域（长江、太湖、黄河、松花江、黑龙江、大运河和东江）^［35］^、巴西桑托斯湾^［36］^、葡萄牙北部海水^［37］^和挪威特罗姆瑟湾^［38］^均已被检测到；其中，葡萄牙北部与本研究区域相似，在夏季检测到高浓度的IBU^［37］^。FPF也是一种广泛使用的非甾体类消炎药，可治疗类风湿性关节炎、退行性关节病、强直性脊柱炎和痛风^［39］^。本研究中FPF夏季浓度显著高于春季和冬季，而土耳其马尔马拉金角湾河口则是秋季浓度显著高于春季和夏季^［40］^，且在马尔马拉金角湾河口中FPF一年四季被检测到。本研究未涉及秋季样本可能是导致上述差异的原因，应在后续研究中对四季样品展开充分研究。PHZ是一种镇痛和解热药物，在临床上广泛用于缓解头痛、发烧和全身疼痛^［41］^，其被确定为烟台湾海域主要污染物^［8］^。PHZ在三季都被检测到可能是因为其生化持久性强，废水处理厂对PHZ的去除效率仅为33%左右^［41］^。本研究中夏季消炎药和总PPCPs夏季浓度显著高于其他两个季节，这与以往的研究结果不同^［8，42，43］^。可能是由于夏季为烟台、威海旅游旺季，大量流动人口带来了非甾体类消炎药消费量和释放量的增长^［44，45］^。此外，夏季采样时间在黄河调水调沙之后，因此污染物浓度还可能受黄河调水调沙的影响，这需要结合悬浮颗粒物上PPCPs浓度数据，以更好地证明调水调沙对海水中污染物浓度的影响，这是未来研究的重要方向。

### 2.3 海水和沉积物中目标PPCPs空间分布特征

在水平方向上，每个采样断面海水中PPCPs总浓度未呈现出明显的离岸由近及远递减的趋势。整体看来，春季和冬季渤海海域采样点（A1~A4）PPCPs总浓度略高于黄海海域采样点（B1~D3），夏季黄海海域采样点中PPCPs总浓度略高于渤海海域采样点（图4）。注入黄海南部莱州湾内的黄河是我国北方最大河流，全年入海水量高达226.50亿立方米^［46］^，是黄海和莱州湾内各类污染物的重要来源。研究表明，在莱州湾主要入海河流中，黄河输入对于莱州湾PPCPs的贡献最大（近90%）^［47］^。随着春季黄河水量的增加，其输入至莱州湾PPCPs污染物的总量增加，导致莱州湾PPCPs浓度高于黄海。渤海海域冬季会出现大量海冰^［48，49］^，导致PPCPs污染物扩散速度大幅度下降。同时大量海冰的存在也会减弱水体中的光照强度，进而导致PPCPs污染物光降解速率下降^［8，50］^，最终使渤海海域采样点冬季PPCPs的浓度变高。夏季黄海采样点PPCPs浓度高可能是因为夏季是旅游旺季，蓬莱、烟台和威海三地是山东省热门旅游城市，大量游客的到来增加了PPCPs的消费量和释放量^［6］^，MPB是常用的化妆品防腐剂，夏季B1点位MPB的质量浓度为638.87 ng/L可以很好地佐证这点。

**图4:**
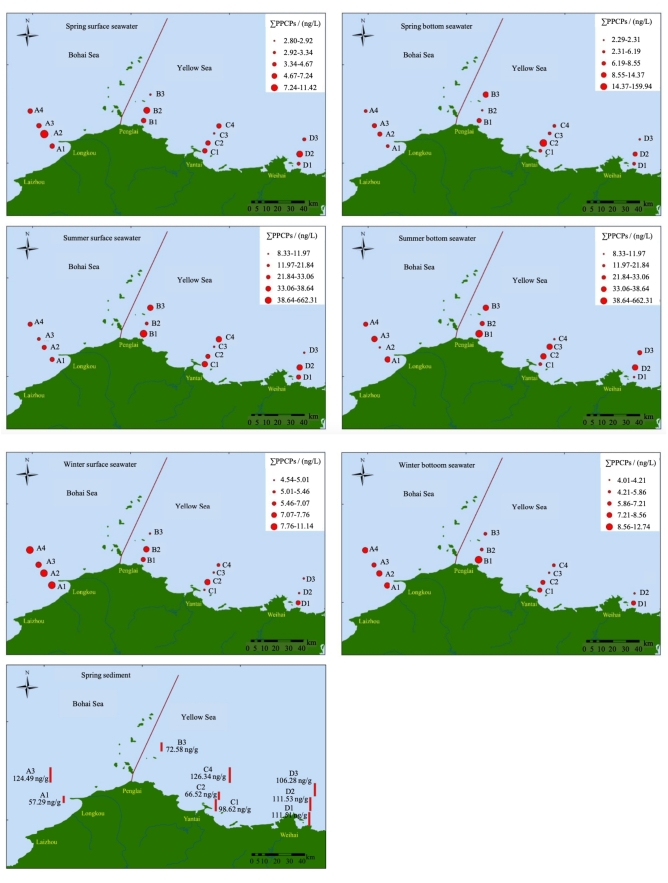
不同季节表底层海水和沉积物中PPCPs总含量的空间分布 The figures were generated using ArcGIS 10.8.

在垂直方向上，虽然春季底层水PPCPs总浓度整体高于表层水，夏季表底层水PPCPs总浓度的分布格局不同，冬季表层水PPCPs总浓度整体高于底层水（图4），但3个季节表层水和底层水PPCPs总浓度并未呈现显著性差异，而且3个季节表底层水非甾体类消炎药和防腐剂的浓度也未呈现出显著性差异（*p*>0.05，图5）。这一结果与此前对于莱州湾海域中抗生素和个人护理品垂向变化的结果一致^［15，51］^。研究表明，污染物的垂向变化特征受其自身理化性质和环境条件的影响^［52，53］^。本研究中目标PPCPs总浓度未呈现显著性差异，可能与海水垂直交换能力强、海水垂直混匀有关^［54-56］^。在沉积物中，PPCPs浓度为黄海点位浓度高于渤海（图4）。需要注意的是，受研究条件限制，本研究中采样点位数量较少，可能在一定程度上限制了研究结果的代表性。未来研究应注意优化采样点数量，以进一步提高研究的全面性和代表性。

**图5:**
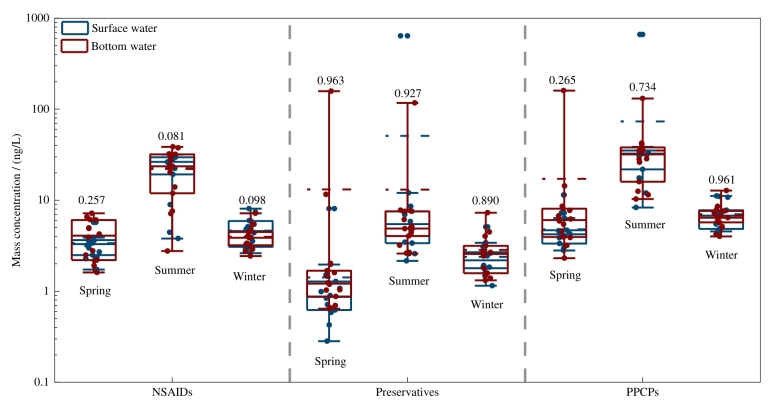
不同季节表底层海水中非甾体类消炎药、防腐剂和PPCPs总浓度对比 Numbers on the boxes represent significant levels between the surface and bottom seawater.

### 2.4 沉积物-水分配系数

污染物在海洋环境中的分布取决于很多因素，例如沉积物-水分配系数^［57］^、辛醇-水分配系数^［57］^、污染物本身性质等，沉积物-水分配系数可提供污染物在海洋环境中迁移和归宿的信息。

沉积物-水分配系数用公式（4）、（5）^［58］^进行计算：


$$K_{d}=C_{s}/C_{w}\times 1000$$ （4）

$$K_{oc}=K_{d}\times 100/f_{oc}$$ （5）

其中*C*_s_和*C*_w_分别为沉积物（ng/g）和水（ng/L）中PPCPs的含量；*f*_oc_为沉积物中总有机碳含量（%）。

本研究所涉及的物质中EPB和PPB的log *K*_oc_值为（3.99±0.95） cm^3^/g和（3.80±0.57） cm^3^/g（图6），只选取这两种物质计算log *K*_oc_值的原因是其他物质未在水中检出或未在沉积物中检出，导致*K*_d_或log *K*_oc_值无法计算。以往研究很少涉及EPB、PPB的沉积物-水分配系数，通过与使用EPIWEB 4.1（US EPA）根据物质理化性质预测的log *K*_oc_值对比发现，本研究中通过野外采样实际测量的log *K*_oc_值均高于其预测值（图6a）。根据以往其他物质的研究，物质的沉积物-水分配系数受物质的性质与用量、水体化学性质和沉积物性质^［59］^等因素影响。在海洋环境中，盐度是影响有机化合物在沉积物-水之间分配行为的重要环境因素，盐度的增加可能导致有机化合物与沉积物间亲和力的改变^［60］^。本次研究中，两种物质log *K*_oc_值和海水pH、盐度及电导率均无显著相关性（*p*>0.05），表明实际环境中有机污染物分配行为的复杂性。未来需进一步深化此方面的研究，以全面阐明环境因素对PPCPs沉积物-水分配行为的影响。

**图6:**
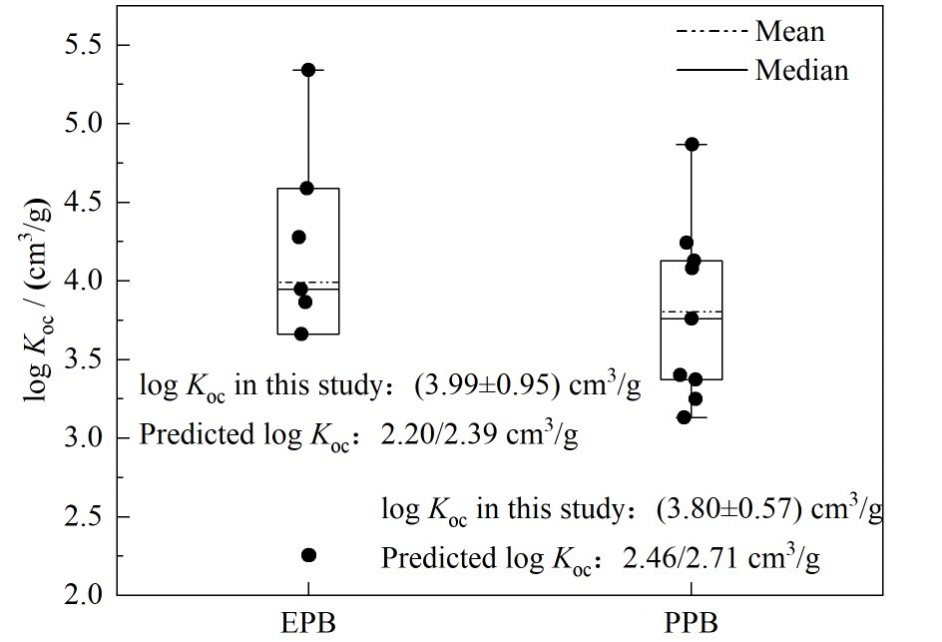
EPB和PPB的沉积物-水分配系数 The predicted log *K*_oc_ is estimated by the Molecular Connectivity Index and log *K*_ow_， respectively.

### 2.5 PPCPs的联合生态风险评估及风险优先级排序

由于PPCPs在海洋环境中是混合存在的，无论毒性机制如何，混合物的毒性可能是所有单个化合物毒性作用之和^［61］^。因此，本研究利用公式（1）分别计算了PPCPs对藻类、甲壳类和鱼类的总风险熵值，以评估PPCPs混合物对不同营养水平水生生物的联合生态风险^［8，15，16，29］^。结果表明，尽管PPCPs混合物对甲壳类的风险高于藻类和鱼类，但其对3类生物均呈低风险（图7a）。这一研究结果与之前马来西亚雪兰莪州^［62］^和天津某水产养殖区^［63］^的研究结果一致，均为低风险；和河套灌区^［64］^的研究结果不一致，河套灌区的研究结果为PPCP物质对生物呈现中高风险，少部分物质呈现低风险，这可能与PPCPs污染物类型和毒性数据选取的差异有关^［15］^。此外，渤海海域采样点中PPCPs混合物对藻类、甲壳类动物和鱼类的生态风险熵均值均高于黄海海域，但两海域间未呈现出显著性差异（*p*>0.05，图7a）。

**图7:**
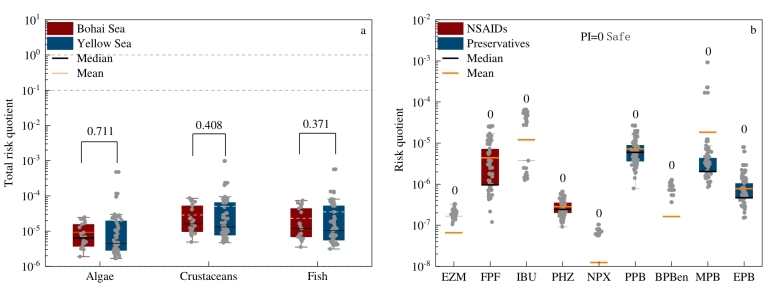
（a）PPCP混合物对不同水生生物的总风险熵（图上数字代表不同海域采样点之间的显著性差异水平）；（b）单一PPCP（检出率高于50%）的风险熵和优先级指数

为对研究区海水中目标PPCPs进行风险优先级排序^［65］^，本研究采用优化的风险熵值法（公式（3））计算了单一PPCP物质（检出率高于50%）对其最敏感物种的优先级指数PI（图7b）。结果表明，所有检出频率大于50%的物质的优先级指数PI均为0，均被归类为安全物质。尽管本研究中非甾体类消炎药和防腐剂的混合物对3类生物的风险均较低，但是对于PPCPs的代谢产物并没有深入研究，同时以往研究^［66］^认为羟基化布洛芬代谢物如1-羟基布洛芬、2-羟基布洛芬、1，2-二羟基布洛芬比母体布洛芬具有更高的毒性，未来仍需对PPCPs及其代谢产物进行持续监测以评估其生态风险。此外，以往该地区的相关研究缺乏对MPB的探讨，因此在后续污染物治理中应着重关注该物质。

## 3 结论

本研究在山东半岛烟威海域探究了两类共15种常用PPCPs的时空分布、沉积物-水分配系数、生态风险及污染物优先级，分别有13种和5种PPCPs在海水和沉积物中被检出，PPCPs在水体和沉积物中的含量分别为2.31~662.31 ng/L和57.29~126.34 ng/g。PPCPs的浓度变化不仅与季节性消费模式有关，也跟污染物自身性质、环境条件的影响有关。在空间变化中，垂直方向上3个季节表层水和底层水并未展现显著性差异，水平方向上春季和冬季渤海海域采样点海水中PPCPs浓度要高于黄海海域采样点，夏季黄海海域采样点PPCPs浓度高于渤海海域采样点，可能与环境条件差异和PPCPs消费模式的变化有关。此外，环境因素可以影响PPCPs的沉积物-水分配系数，进而影响其环境归趋。生态风险方面，各类PPCPs对藻类、甲壳类和鱼类均呈低风险，9种PPCPs优先级指数均为0，但是MPB在3个点位的风险熵值高于其他物质和其他点位，所以在未来的研究中，有必要对该区域的MPB污染物着重关注，并研究其去除方式。

## References

[1] Wang W， Hou Y J， Shi Y Z， et al. Acta Laboratorium Animalis Scientia Sinica， 2024， 32（8）： 1084

[2] Li H W， Wei G Z， Dai Y . Flavour Fragrance Cosmetics， 2024（3）： 212

[3] Yu Y， Li W， Lu S， et al. Ecotox Environ Safe， 2019， 182： 109419 10.1016/j.ecoenv.2019.109419 31301591

[4] Klosterhaus S L， Grace R， Hamilton M C， et al. Environ Int， 2013， 54： 92 23527629 10.1016/j.envint.2013.01.009

[5] Ali A M， Ronning H T， Alarif W， et al. Chemosphere， 2017， 175： 505 28249192 10.1016/j.chemosphere.2017.02.095

[6] Chen H， Chen W， Guo H， et al. Environ Sci Pollut Res， 2021， 28（18）： 22716 10.1007/s11356-020-12335-1 PMC7797026 33423193

[7] Guo X， Lv M， Song L， et al. Environ Sci Technol， 2023， 57（51）： 21823 38078887 10.1021/acs.est.3c06522

[8] Guo X， Lv M， Song L， et al. J Hazard Mater， 2023， 459： 132163 37515990 10.1016/j.jhazmat.2023.132163

[9] Chen X， Lei L， Liu S， et al. Sci Total Environ， 2021， 792： 148352 34147798 10.1016/j.scitotenv.2021.148352 PMC8197610

[10] Kang R， Zhou S Y， Chen T T， et al. Water， 2024， 16（7）： 17

[11] Wu Z， Liu L L， Zhang X X， et al. Mar Pollut Bull， 2023， 190： 8 10.1016/j.marpolbul.2023.114885 37015173

[12] Li F， Ma Y Q， Song X K， et al. Front Mar Sci， 2022， 9： 14

[13] Li P M， Gao X L . Ecotox Environ Safe， 2014， 109： 1 10.1016/j.ecoenv.2014.07.023 25128644

[14] Yu X P， Yu F R， Li Z P， et al. J Hazard Mater， 2023， 443： 12 10.1016/j.jhazmat.2022.130369 36444065

[15] Lu S， Lin C， Lei K， et al. J Hazard Mater， 2022， 424（Pt B）： 127487 34655873 10.1016/j.jhazmat.2021.127487

[16] Pusceddu F H， Choueri R B， Pereira C D S， et al. Environ Pollut， 2018， 232： 274 28958726 10.1016/j.envpol.2017.09.046

[17] Du B H， Fan G D， Yu W W， et al. Environ Pollut， 2020， 267： 13 10.1016/j.envpol.2020.115405 32866865

[18] Liu S S， Zhao H X， Lehmler H J， et al. Environ Sci Technol， 2017， 51（4）： 2392 28106989 10.1021/acs.est.6b04556 PMC5618103

[19] Lu G H， Piao H T， Gai N， et al. Arch Environ Contam Toxicol， 2019， 76（2）： 255 30390119 10.1007/s00244-018-0578-y

[20] Zheng Y， Lu G H， Shao P W， et al. Arch Environ Contam Toxicol， 2020， 78（4）： 579 32123946 10.1007/s00244-020-00725-y

[21] Yang L， Zhou Y Q， Shi B， et al. Environ Int， 2020， 135： 10 10.1016/j.envint.2019.105306 31881428

[22] Han Q F， Song C， Sun X， et al. Chemosphere， 2021， 279： 130381 33878699 10.1016/j.chemosphere.2021.130381

[23] Lin K， Wang R， Han T， et al. Sci Total Environ， 2023， 857（Pt 3）： 159682 36302405 10.1016/j.scitotenv.2022.159682

[24] Duarte D J， Oldenkamp R， Ragas A M J . Integr Environ Asses， 2022， 18（6）： 1639 10.1002/ieam.4588 PMC9790459 35112470

[25] Jurado A， Vázquez-Suñé E， Pujades E . Water， 2021， 13（5）： 720

[26] de Jongh C M， Kooij P J， de Voogt P， et al. Sci Total Environ， 2012， 427/428： 70 22551934 10.1016/j.scitotenv.2012.04.010

[27] Fonseca E， Hernandez F， Ibanez M， et al. Environ Int， 2020， 144： 106004 32745782 10.1016/j.envint.2020.106004

[28] Derisso C R， Pompei C M E， Spadoto M， et al. Water Air Soil Poll， 2020， 231（9）： 468

[29] Pei S， Li B， Wang B， et al. Water， 2022， 14（13）： 1999

[30] Sadutto D， Andreu V， Ilo T， et al. Environ Pollut， 2021， 271： 11 10.1016/j.envpol.2020.116353 33385890

[31] Yang Y， Fu J， Peng H， et al. J Hazard Mater， 2011， 190（1-3）： 588 21497014 10.1016/j.jhazmat.2011.03.092

[32] Lv M， Sun Q， Hu A Y， et al. J Hazard Mater， 2014， 280： 696 25232652 10.1016/j.jhazmat.2014.08.054

[33] Emnet P， Mahaliyana A S， Northcott G， et al. Arch Environ Contam Toxicol， 2020， 79（4）： 461 33128586 10.1007/s00244-020-00760-9

[34] Jonkers N， Sousa A， Galante-Oliveira S， et al. Environ Sci Pollut Res， 2010， 17（4）： 834 10.1007/s11356-009-0275-5 PMC2854360 20017000

[35] Du Y D， Zhang X Q， Shu L， et al. Environ Sci Pollut Res， 2021， 28（30）： 40568 10.1007/s11356-020-09714-z 32564323

[36] Pereira C D S， Maranho L A， Cortez F S， et al. Sci Total Environ， 2016， 548： 148 26802343 10.1016/j.scitotenv.2016.01.051

[37] Lolic A， Paíga P， Santos L， et al. Sci Total Environ， 2015， 508： 240 25481252 10.1016/j.scitotenv.2014.11.097

[38] Weigel S， Berger U， Jensen E， et al. Chemosphere， 2004， 56（6）： 583 15212901 10.1016/j.chemosphere.2004.04.015

[39] Useini L， Mojic M， Laube M， et al. ChemMedChem， 2023， 18（5）： 8 10.1002/cmdc.202200583 36583943

[40] Korkmaz N E， Caglar N B， Aksu A . Reg Stud Mar Sci， 2022， 51： 13

[41] Miao H F， Cao M， Xu D Y， et al. Chemosphere， 2015， 119： 326 25038548 10.1016/j.chemosphere.2014.06.082

[42] Jiang X S， Zhu Y Q， Liu L Q， et al. Sci Total Environ， 2021， 762： 10 10.1016/j.scitotenv.2020.143138 33121774

[43] Yang L， He J T， Su S H， et al. Environ Sci Pollut Res， 2017， 24（18）： 15838 10.1007/s11356-017-8999-0 28534270

[44] Adeleye A S， Xue J， Zhao Y X， et al. J Hazard Mater， 2022， 424（Part： B）： 127284 34655870 10.1016/j.jhazmat.2021.127284

[45] Ren B N， Geng J， Wang Y， et al. Environ Geochem Hlth， 2021， 43（8）： 3083 10.1007/s10653-021-00828-y 33502681

[46] Yellow River Conservancy Commission of the Ministry of Water Resources . 2023 Yellow River Water Resources Bulletin

[47] Lu S . ［PhD Dissertation］. Beijing： Beijing Normal University， 2022

[48] Guo Y D， Wang T， Cheng S J， et al. Remote Sensing for Natural Resources， 2024， 36（1）： 242

[49] Yin H J， Liu T， Chen X， et al. Hydro Science and Cold Zone Engineering， 2023， 6（10）： 1

[50] Carlson J C， Stefan M I， Parnis J M， et al. Water Res， 2015， 84： 350 26282501 10.1016/j.watres.2015.04.013

[51] Lu S， Wang B D， Xin M， et al. Sci Total Environ， 2022， 810： 152290 34902407 10.1016/j.scitotenv.2021.152290

[52] Liang X M， Chen B W， Nie X P， et al. Chemosphere， 2013， 92（11）： 1410 23628172 10.1016/j.chemosphere.2013.03.044

[53] Liu X H， Zhang H B， Li L Z， et al. Mar Pollut Bull， 2016， 109（1）： 597 27245555 10.1016/j.marpolbul.2016.05.033

[54] Han Y J . ［MS Dissertation］. Jinan： Shandong University， 2020

[55] Xin M . ［MS Dissertation］. Qingdao： First Institute of Oceanography， Ministry of Natural Resources， 2011

[56] Yang B， Gao X L， Xing Q G . Cont Shelf Res， 2018， 171： 113

[57] Fairbairn D J， Karpuzcu M E， Arnold W A， et al. Sci Total Environ， 2015， 505： 896 25461092 10.1016/j.scitotenv.2014.10.046

[58] Jin H B， Zhu L Y . Water Res， 2016， 103： 343 27486043 10.1016/j.watres.2016.07.059

[59] Drillia P， Stamatelatou K， Lyberatos G . Chemosphere， 2005， 60（8）： 1034 15993150 10.1016/j.chemosphere.2005.01.032

[60] Zhu J Q， Guo R Y， Ren F F， et al. Sci Total Environ， 2024， 914： 170046 38218485 10.1016/j.scitotenv.2024.170046

[61] US EPA . Supplementary Guidance for Conducting Health Risk Assessment of Chemical Mixtures.（2000-08-31） ［2025-03-11］. http://cfpub.epa.gov/ncea/risk/recordisplay.cfm?deid=20533 http://cfpub.epa.gov/ncea/risk/recordisplay.cfm?deid=20533

[62] Hanafiah Z M， Mohtar W， Abd Manan T S， et al. PeerJ， 2023， 11： 28

[63] Wu Y Q， Song S， Chen X C， et al. Sci Total Environ， 2023， 854： 11 10.1016/j.scitotenv.2022.158792 36113789

[64] Wang C， Lu Y L， Sun B， et al. Sci Total Environ， 2022， 851： 9 10.1016/j.scitotenv.2022.158179 35988592

[65] Zhou S B， Di Paolo C， Wu X， et al. Environ Int， 2019， 128： 1 31029973 10.1016/j.envint.2019.04.034

[66] Rastogi A， Tiwari M K， Ghangrekar M M . J Environ Manage， 2021， 300： 20 10.1016/j.jenvman.2021.113694 34537557

